# Epidemiology of Geriatric-Dermatology virtual clinic: Teledermatology-Based care for elderly patients with skin diseases in Qatar

**DOI:** 10.5339/qmj.2023.sqac.12

**Published:** 2023-11-19

**Authors:** Sara Al-Khawaga, Wasim Akram, Khairunnisa Hussain, Mani Chandran, Joerg Buddenkotte, Febu Elizabeth Joy, Sena Manjooran, Basheer Kolaparambath, Hala Mosaad, Khalifa Al-Naama, Hanadi AL Hamad, Martin Steinhoff

**Affiliations:** ^1^Department of Dermatology and Venereology, Hamad Medical Corporation, Doha, Qatar Email: MSteinhoff@hamad.qa; ^2^Dermatology Institute, Academic Health System, Hamad Medical Corporation, Doha, Qatar; ^3^Translational Research Institute, Academic Health System, Hamad Medical Corporation, Doha, Qatar; ^4^Weill Cornell Medicine-Qatar, Doha, Qatar; ^5^Geriatrics Department, Hamad Medical Corporation, Doha, Qatar; ^6^Qatar University, College of Medicine, Doha, Qatar; ^7^Department of Dermatology, Weill Cornell Medicine, New York, USA; ^8^College of Health and Life Sciences, Hamad Bin Khalifa University-Qatar

## Abstract

**Background:** The ‘GeriDerm’ (geriatric dermatology) clinic, is a new dermatology-based service at Hamad Medical Corporation (HMC), accommodating the needs of our elderly population living in the State of Qatar. Due to the global demographic transition towards an elderly population (≥65 years of age), incidences of chronic diseases, including dermatologic conditions, rise in parallel. Patients of older age are at higher risk of using multiple medications, seeing multiple care providers, often receiving multiple diverging pieces of information, and feeling lost within the system. Taking into consideration the elderly unique characteristics, the Geriatric Dermatology telemedicine clinic is a novel approach to meeting the many challenges our elderly patients face via providing quick, accurate assessments of cognition, functional status, frailty screening, and assessment for polypharmacy.

**Methods:** Data of 1080 elderly patients with various skin disorders from June 2020 to July 2021 was received from the Dermatology Geriatric clinic, and then reviewed.

**Results:** There were 521(48.2%) new cases and 559(51.8%) follow-up cases who attended the clinic either virtually or face to face consultation. A total of 587(54.4%) female and 493(45.6%) male elderly patients attended the clinic. The mean age was 74.6, with a minimum age of 60 and a maximum age of 106 years. 57.9%(625) of GeriDerm patients were Qatari, followed by Palestinian 75(6.9%), Syrian 51(4.7%), Egyptian 46(4.3%), and Indian 44(4.1%); while other nationalities constituted 239(22.1%). The majority of the cases were Contact Dermatitis 146(13%), Bullous Pemphigoid 107 (10%), and Pruritis 101(9.4%).

**Conclusion:** The ‘GeriDerm’ service at HMC aimed to achieve the best healthcare standards for the elderly population of Qatar during COVID-19 pandemic, and is now established as a continuous advanced technology-based framework facilitating caring for older patients with skin disease via providing a clear pathway for adequate triaging, identification of severe conditions (red flag) requiring in-person clinic visits, while managing non-life threatening dermatoses via a teledermatology based approach.

